# Acceptability of Plant-Based Diets for People with Chronic Kidney Disease: Perspectives of Renal Dietitians

**DOI:** 10.3390/nu14010216

**Published:** 2022-01-04

**Authors:** Jordan Stanford, Mikaela Zuck, Anita Stefoska-Needham, Karen Charlton, Kelly Lambert

**Affiliations:** 1 School of Medicine, Faculty of Science, Medicine and Health, University of Wollongong, Northfields Ave, Wollongong, NSW 2522, Australia; mz849@uowmail.edu.au (M.Z.); anitasn@uow.edu.au (A.S.-N.); karenc@uow.edu.au (K.C.); klambert@uow.edu.au (K.L.); 2 Illawarra Health and Medical Research Institute, Wollongong, NSW 2522, Australia

**Keywords:** plant-based diets, chronic kidney disease, implementation, barriers, enablers, cross-sectional survey, qualitative research

## Abstract

The purpose of this study was to explore the perspective of renal dietitians regarding plant-based diets for chronic kidney disease (CKD) management and evaluate the acceptability of a hypothetical plant-based dietary prescription aiming for the consumption of 30 unique plant foods per week. This study used an exploratory mixed methods design. Forty-six renal dietitians participated in either an online survey (*n* = 35) or an in-depth interview (*n* = 11). Dietitians perceived that plant-based diets could address multiple clinical concerns relevant to CKD. Forty percent of survey respondents reported the hypothetical dietary prescription was realistic for people with CKD, 34.3% were unsure, and 25.7% perceived it as unrealistic. Strengths of the hypothetical prescription included shifting the focus to whole foods and using practical resources like recipes. Limited staffing, time, and follow-up opportunities with patients, as well as differing nutrition philosophies were the most commonly reported challenges to implementation; while a supportive multidisciplinary team was identified as an important enabler. To increase patient acceptance of plant-based dietary approaches, education about plant food benefits was recommended, as was implementing small, incremental dietary changes. Successful implementation of plant-based diets is perceived to require frequent patient contact and ongoing education and support by a dietitian. Buy-in from the multidisciplinary team was also considered imperative.

## 1. Introduction

Diet plays a central role in the management of chronic kidney disease (CKD) [1]. However, dietary prescriptions are often confusing and divergent from standard healthy eating guidelines and may limit healthy plant foods such as fruits, vegetables, and whole-grain products due to their potassium content [2]. Such restrictive dietary guidance has broader implications for people living with CKD by resulting in limited intakes of health-protective food components such as dietary fibre and phytochemicals, as well as potentially contributing to poor dietary adherence overall. Concerns about the contribution of potassium from plant-based foods may be outdated given the emerging evidence that suggests the bioavailability of potassium in whole non-processed fruits and vegetables are lower than initially estimated [3], at around 50–60% [4]. A recent study confirmed that dietary potassium intake was not associated with hyperkalaemia or death in patients receiving haemodialysis treatment [5]. Research indicates that habitual dietary patterns rich in plant-based sources are protective against disease progression and risk of mortality in people with CKD, even at advanced disease stages [6,7]. Additionally, there is growing recognition of the role of plant-based diets in modulating the composition and metabolic activity of the human gut microbiome, which in turn may lead to improved health outcomes relevant to individuals with CKD [8,9,10,11].

In light of this evidence, a less didactic and more liberal educational approach to the renal diet may be possible for patients with CKD. However, successful implementation of novel diet therapies in clinical practice requires acceptance from practitioners before making them available to their patients. Dietitians provide extensive education to patients, caregivers, and their families to facilitate appropriate food choices and improve long-term dietary adherence, which may alleviate disease progression [2]. Additionally, dietitians have unique first-hand insights into the challenges faced by patients regarding the prescription of a complex therapeutic diet [12]. Therefore, the objectives of this study were to explore renal dietitians’ perspectives regarding plant-based diets for CKD management and evaluate the acceptability of a hypothetical plant-based dietary prescription and accompanying print resources. The hypothetical dietary prescription, which aimed to increase the amount and variety of plant foods in diets for people with CKD, was used to stimulate discussion and facilitate recommendations for implementing plant-based diets in future clinical trials and current practice.

## 2. Materials and Methods

This exploratory mixed-methods study was approved by the joint Illawarra Shoalhaven Local Health District/University of Wollongong Human Research Ethics Committee (2019/ETH00397). The Checklist for Reporting Results of Internet E-Surveys [13] was used as a guide to create the online survey. The consolidated criteria for reporting qualitative research (COREQ) checklist (Item S1, Supplementary Materials) was used to facilitate a detailed and comprehensive reporting of the qualitative component of the study [14].

### 2.1. Study Sample and Recruitment

Accredited Practising Dietitians (APDs) or those eligible for APD status actively working in primary, secondary, or tertiary care employed to provide dietary advice to people with CKD in Australia were eligible to take part in this study. A convenience sample of renal dietitians was recruited using two approaches to provide the greatest possible coverage and to maximise the participation of the specialist target group [15]. Recruitment took place between April and August 2019, where eligible dietitians were contacted via: (i) a professional e-mail distribution network for renal dietitians in Australia; and (ii) attendance at the 2019 World Congress of Nephrology Renal Nutrition, Nursing, and Allied Health Professionals Symposium. Conference attendees were identified with the assistance of conference organisers. Eligible dietitians were invited to participate by e-mail or in person and given the option to partake in either an in-depth, semi-structured interview, or complete a short online survey. Advertising materials included an overview of the study, a participant information sheet with investigators’ contact details, interview questions, and a direct URL link to the online survey. Participants wanting to participate in an interview rather than the survey were advised to contact one of the investigators to schedule a meeting. No incentives were offered, and participation was voluntary.

### 2.2. Hypothetical Plant-Based Dietary Prescription

Survey and interview participants were informed that, in the context of this study, a ‘plant-based dietary prescription’ referred to a diet dominated by a variety of vegetables, fruits, legumes, whole grains, nuts, seeds, herbs, and spices. It did not infer that the eating pattern was exclusively vegetarian or vegan and could include small to moderate amounts of animal-based products such as dairy, meats, poultry, and fish. The hypothetical plant-based dietary prescription designed by members of the research team aimed to increase the amount and variety of plant foods in the diets of people with CKD. The philosophy to achieve a plant-based diet introduced to all participants in this study was simplified by encouraging patients to consume 30 or more unique plant foods over a seven-day period. This concept was informed by the findings of the largest observational study to date investigating the human gut microbiome [16], whereby individuals consuming a higher plant-based diet (defined as consuming more than 30 different varieties of plant foods per week) had increased microbial diversity and lower antibiotic-resistant microbial genes [16].

Further details about how the hypothetical prescription could be implemented with patients in practice were explored with interview participants exclusively. For instance, to supplement the target of consuming 30 or more unique plant foods per week, specific advice was proposed about the number of daily food servings patients would need to consume to more closely align to the Australian Dietary Guidelines (i.e., five servings of vegetable, two servings of fruits, etc.) [17], while still adhering to the evidence-based guidelines for nutrition in kidney disease [18,19]. Five ancillary print resources were also developed to accompany the hypothetical plant-based dietary prescription. The print resources included a recipe book, a seven-day template for participants to fill out and plan meals, an A-Z food guide to build meals, a food swap list, and an instruction manual on how these resources could be used.

### 2.3. Data Collection and Analysis

The anonymous online survey consisted of eight questions (Item S1, Supplementary Materials), including close-ended questions (multiple-choice) and open-ended questions, which were pilot-tested with three dietitians to assess face validity. Multiple responses were accepted for questions 2, 4, 5, and 6. This online survey was self-administered using SurveyMonkey (San Mateo, CA, USA) and was open for 10 weeks from 26 June 2019, to 4 September 2019. Tacit consent was implied by online survey completion. Participants were only able to complete the survey once and could review and change their answers on any survey page until they submitted the survey. Analysis of quantitative data was facilitated using Microsoft Excel (2010). Data presented in figures were produced using R version 3.5.0 [20]. Open-ended responses were analysed using deductive content analysis [21]. Only participants with complete survey data were included.

Semi-structured interviews, conducted by two research members, lasted between 30–45 min and were undertaken either face-to-face, via Skype^®^ or telephone. The semi-structured interview guide covered four key topic areas (Item S3, Supplementary Materials, Item S3). For those interviewed by phone, the print resources were emailed before the interview once written consent had been obtained. Demographic details (age, gender, length of practice as a dietitian, length of practice as a renal dietitian, current full-rime equivalent (FTE) load, practice setting) were collected. Interviews were recorded using a digital recorder and transcribed verbatim. Dedoose software was used to manage and code the data [22]. Transcripts were analysed inductively using Braun and Clarke’s six phases of thematic analysis [23]. Specifically, two researchers read and reread the transcribed verbatim independently for immersion in the data. Quotes relevant to the research question were highlighted, and codes were systematically applied to identify elements of interest. Codes were collated into potential subthemes. The inter-reliability of codes was examined in a subset of transcripts by a third member of the research team to ensure the credibility of the coding analysis. Researchers worked collaboratively to reach a consensus on the key themes that emerged in the assigned codes and subthemes. Ongoing analysis took place to refine each key theme to ensure that they were reflective of the coded extracts and the entire data set. Compelling extracts were selected for final analysis, relating back to the research question and literature. Participants did not provide feedback on the key themes.

## 3. Results

Approximately 120 renal dietitians were invited to take part in this study, and 46 (response rate: 38.3%) either completed the online survey (*n* = 35) or participated in an in-depth interview (*n* = 11).

### 3.1. Online Survey

The median duration of time the 35 survey respondents currently worked as renal dietitians was seven years (Table 1). The case mix of patients seen by the survey participants was dominated by those receiving haemodialysis (*n* = 31, 88.6%) and patients in the pre-dialysis stage (*n* = 23, 65.7%).

**Figure 1:**
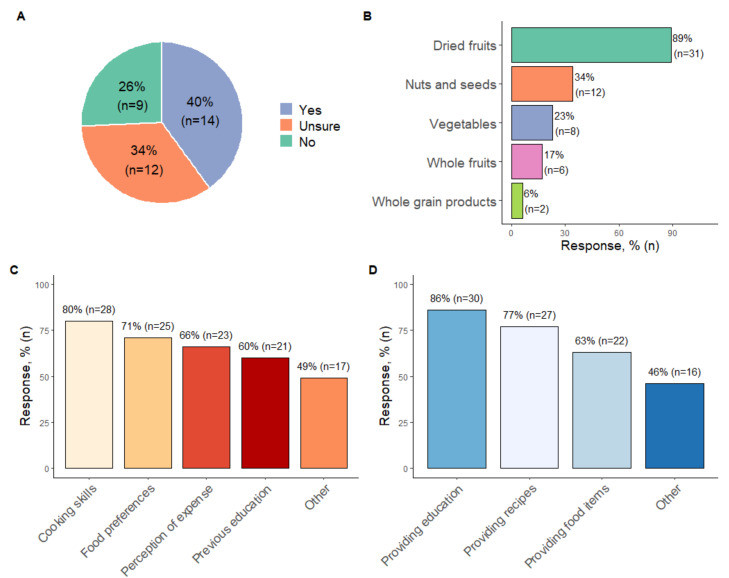
Summary of responses to multiple-choice survey questions. (**A**) Answers to whether the target of consuming 30 unique plant-based foods over seven days is realistic for patients with CKD. (**B**) Responses to the question about which plant foods dietitians felt cautious about prescribing to people with CKD. (**C**) Potential challenges to implementing plant-based diets for people with CKD. (**D**) Potential enablers to implementing plant-based diets for people with CKD. Multiple responses were accepted for the questions presented in figures (**B**–**D**).

Forty percent of survey participants reported that the proposed hypothetical dietary prescription recommending consumption of 30 unique plant-based foods per week was realistic for people with CKD (Figure 1A). The following quotes echo the most common justifications provided: “this amount reflects the healthy eating guidelines”; “I have already had success in implementing this in my role as a renal dietitian”, and “when breaking it down into 4–5 different plant foods per day from a variety of sources, it [the hypothetical plant-based dietary prescription] doesn’t seem excessive”. However, support was not unanimous, and several participants expressed that they were unsure (*n* = 12, 34.3%) or felt the hypothetical plan was unrealistic (*n* = 9, 25.7%). For example, “misinformation provided by other health care professionals”, “financial burdens”, and “limited accessibility to fresh foods” were concerns impacting implementation.

Lack of cooking and preparation skills were also considered substantial barriers to implementation, in addition to personal food preferences (Figure 1B). Other barriers identified were “managing patients’ fear around potassium control” (*n* = 17, 48.57%). Furthermore, when implementing plant-based diets for patients with CKD, renal dietitians consistently reported they would be cautious about prescribing more dried fruit, followed by nuts and seeds (Figure 1C). The main reason for using a cautious approach to these items was fear of inducing hyperkalaemia and/or hyperglycaemia. Suggestions to enhance implementation included educating patients about the health benefits of plant-based eating and providing recipes (Figure 1D). Other recommendations included “gaining support from the multidisciplinary team”, “education with motivated patients at earlier stages of CKD”, and “availability of supplementary educational materials such as food checklists, meal plans, and pictorial resources”.

### 3.2. In-Depth Interviews

Twelve renal dietitians expressed interest, of which eleven participated in an in-depth interview. Non-participation was due to scheduling conflicts. Data saturation was reached by the 11th interview, with no new themes subsequently identified. All interview participants were female (age range 25–64 years). Participants worked in various geographic locations, with diverse CKD populations and varying levels of renal dietetic experience (Table 1). Two overarching themes from the interviews were: (i) the value of plant-based diets and strengths of the hypothetical dietary prescription; and (ii) existing barriers and enablers to successful implementation. A further eight sub-themes were apparent.

#### 3.2.1. Value of Plant-Based Diets and Strengths of the Hypothetical Dietary Prescription

##### Addresses Multiple Clinical Concerns

All interviewees acknowledged that a plant-based diet could prevent or manage various risk factors for disease progression, including comorbidities relevant to individuals with CKD.

“…so if they are eating this way…glycemic control will be better, hypertension control will be better, proteinuria is likely to be better, …all of the things that you would be worried about.. a plant-based diet is going to help with that”

(Dietitian 11)

##### Shifts Focus from Nutrients to Whole Foods

Participants repeatedly indicated that traditional approaches to dietary counselling for CKD, which tend to focus on individual nutrients, can lead to patients feeling confused. Respondents discussed that a focus on nutrients does not consider overall diet quality and often results in whole foods, like fruits and vegetables, being erroneously restricted or removed by patients because they are rich sources of potassium.

“We’re very focused on guidelines and millimoles of potassium, but I get concerned … when I provide my education…that people are not able to put that into practice or they get the message wrong and then end up cutting out things unnecessarily because they think it’s bad for them…It [the hypothetical dietary prescription] focuses on making healthy food choices because I think people are misunderstanding the information about potassium and phosphate and see it as cutting out fruit and vegetables…and that is a very big concern…When in fact you want to maintain a healthy diet overall and variety… it [the hypothetical dietary prescription] allows for flexibility, and it allows people to transition from receiving some abstract information about food into this is what I’m going to eat today.”

(Dietitian 10)

Dietitians in this study perceived that using a simple target such as ‘30 unique plant foods per week’ might help alleviate patient confusion. Respondents also felt that the increased focus on foods rather than nutrients in the hypothetical dietary prescription would likely improve overall diet quality. Positive dietary messaging used in the hypothetical prescription was preferred (i.e., include these foods) rather than negative messaging (i.e., limit consumption of these foods) as a strategy to reduce patient anxiety and encourage adherence to dietary recommendations.

##### A Need for Practical Complementary Resources

Additional strengths of the hypothetical dietary prescription were the inclusion of recipes and the food swap list. These resources were perceived to be valuable tools to help demonstrate how various plant foods could be incorporated into meals, aid in building customisable meal plans, and accommodate individual food preferences.

“The recipes are a great suggestion because there is no point giving this if people don’t have the skills or the knowledge of how to incorporate those foods to make it into a meal… demonstrating it is achievable.”

(Dietitian 5)

“I like the variety… they have this list of foods that they can swap, like each meal, each fruit serve out with, so that really helps.”

(Dietitian 5)

#### 3.2.2. Barriers and Enablers to Implementation

##### Organisational Norms and System Inadequacies Are Barriers

Dietitian participants reported that tensions heightened by larger and more significant organisational norms and system inadequacies presented deterring barriers. For example, limited time compounded by limited staffing resources may often result in the inadequate follow-up of patients, which were expressed as common barriers in existing practice and suspected to be problematic, particularly for implementing plant-based diets in practice.

“I think it would be crucial about our follow up … in our pre-dialysis clinic, we do not see patients for like another two to three months, just because of our wait times…”

(Dietitian 7)

“Generally, dietitians do not have the time to do these sorts of detailed meal plans.”

(Dietitian 3)

“...you would need to spend a fair bit of time making sure that the patients understand and checking it.”

(Dietitian 2)

The dietitians stressed the importance of having supportive systems (such as adequate staffing and time) in place that are conducive to ongoing dietetic review and patient monitoring. Respondents suggested this as essential to ensure patient safety, monitor the overall nutritional adequacy of the diet (avoiding any nutrient deficiencies), and support patients to implement dietary recommendations as intended.

##### Differing Nutrition Philosophies and Perceptions about Diet Are Barriers

Similar to the survey respondents, there was noted to be differences in philosophies by some medical and nursing colleagues about the ‘renal diet’. This was identified to be a major obstacle to implementation. Dietitians reported that some other members of the multidisciplinary team (MDT) may provide overly restrictive dietary advice and do not consider the quality of overall dietary patterns, nutrient bioavailability, nor acknowledge other non-dietary causes of electrolyte abnormalities relevant to people with CKD.

“On this sort of diet [high plant-based diet] … that perception, that they have to restrict them because of the potassium, and doctors or people, other people who might also have that perception and not, who do not sort of understand the difference in bioavailability.”

(Dietitian 8)

“Depending on the medication they’re on, they can be more susceptible to hyperkalaemia regardless of what they’re eating...”

(Dietitian 11)

The importance of shifting philosophies and perspectives regarding the renal diet was not limited to the MDT but also the patients themselves. Conflicting sources of information, in addition to previous dietary advice, were perceived to impact patient willingness to adopt new dietary recommendations.

“You have a patient who is very compliant with previous dietary recommendations; they often do not like to go against that.”

(Dietitian 4)

“Trying to move towards [a plant-based diet]…which will undoubtedly include some of the foods that a lot of them just will not touch. It has been entrenched in them … I cannot eat that sort of food…”

(Dietitian 7)

##### Supportive Multidisciplinary Networks Facilitates Implementation

Collaboration with MDT members to ensure consistency of messaging about plant-based diets was considered to be essential for translating plant-based diets into clinical practice. Interviewees suggested that regular in-services, team meetings, or journal clubs with the MDT may be helpful to disseminate and discuss up-to-date nutritional literature.

“[Implementation] would [need] convincing other health staff about the research because many others other than dietitians provide dietary education...you have got doctors…or nurses. So making sure they are aware of the evidence …because …if a patient hears something from their doctor, they [the patient] will listen to them over what we [dietitians] recommend so, I think making sure that the message is consistent.”

(Dietitian 4)

##### Timing of Implementation

Dietitians suggested that careful consideration should be given to the patient’s overall treatment plan when judging an appropriate time to implement a plant-based dietary approach. Like survey respondents, many interviewees agreed that a plant-based diet would most benefit individuals in earlier stages of CKD in terms of clinical outcomes, safety, and patients might be more motivated by preventative measures to delay the initiation of renal replacement therapies.

“[if] they are preparing for dialysis… patients can be quite overwhelmed …, so I think [managing] the complexity of the diet you are trying to prescribe, but also managing their cognitive and emotional states as well can be quite challenging.”

(Dietitian 11)

“… they’re unlikely to have seen a dietitian at CKD stage 3, so you know they don’t have all those restrictions placed on them…they typically are more motivated as they don’t want dialysis and are more likely to benefit from using nutrition as a preventative to delay disease progression…I think if you can get them early, then I think that’s wonderful.”

(Dietitian 1)

##### ‘Marketing’ the Plant-Based Approach

A recommended strategy to enhance implementation was to “market” the plant-based diet to patients. It was suggested that the benefits of the diet should be explained clearly in the first session to motivate patients and encourage adherence, especially for those patients who are asymptomatic at the time of education.

“I think if we are very clear on the outcomes we are looking at, we could sell this to patients…what outcomes can we expect because if people know that it might actually maintain their kidney function for another two years, or three years, or five years, that could be a very big motivator for them. But if we are just talking about general health, they already feel okay, especially when they are in the earlier stages.”

(Dietitian 11)

Furthermore, a graduated goal system to encourage patients to achieve the target of 30 different plant foods over time rather than immediately was also recommended to improve implementation. Improvements to the presentation of dietary targets (30 unique plant foods) in the accompanying print resources of the hypothetical prescription were suggested.

“… a starting point of …I am having x amount of plant-based foods… over the next two weeks can I increase it by an extra 5 or 10 foods a week…so it can be a little bit more of a step guided approach ... that could be another strategy to help them with goal setting.”.

(Dietitian 11)

Several suggested improvements to the supplementary print resources were also offered. Respondents felt that the format of the seven-day template (Item S4, Supplementary Materials) should provide greater detail by including headings to break down into individual meals and snacks for each day. Providing a separate column for ‘oils and spreads’ instead of grouping these products in with the ‘extras’ category, and using standardised serving sizes for each food group listed on the swap list (Item S5, Supplementary Materials) were other recommendations. Participants also emphasised that the resources provided need to be attentive to the health literacy levels of patients and should consider including more pictures.

“Having it … mapped out …how many meals they like to have over the day and being able to fit it in that way… some people might find it a little bit easier to …actually to see how it fits into … breakfast, lunch and dinner, or how many meals a day.”

(Dietitian 11)

“I think it’s good how you have under free vegetables you say one serve is one cup or half a cup cooked. I think its nice to have a standard serve for as many of the foods within a category.”

(Dietitian 10)

“Pictures…. I think pictures are really important …particularly with literacy and also non-English speaking patients.”

(Dietitian 7)

## 4. Discussion

Renal dietitians were aware that encouraging a plant-based diet could benefit individuals with CKD and translate to favourable clinical outcomes. Successful implementation of plant-based diets was perceived to require frequent patient contact and ongoing education and support by a dietitian. Common concerns regarding the use of plant-based diets such as high potassium intakes and protein inadequacy could be alleviated with regular dietetic input. Several studies involving people with or without CKD have demonstrated more than adequate levels of total protein consumption in various types of plant-based diets, including those adhering to a vegan dietary pattern [24]. Although, plant proteins may have insufficient levels of one or more essential amino acids. A recent modelling study [25] found that low-protein, plant-based diets did not meet the recommended dietary allowance for all essential amino acids, reinforcing the need for careful planning and dietetic supervision to ensure the adequacy of all nutrients in the diet. However, some barriers to translation into current practice were identified. The lack of staffing, capacity, and time outlined by renal dietitians in this study is consistent with previous research in North America [26,27,28], Australia [29], and the United Kingdom [30]. New models of dietetic care may be necessary, as it has been demonstrated that patients seen by renal dietitians have fewer hospitalisations and are associated with delays in dialysis commencement [31]. In this study, dietitians highlighted that a coordinated multidisciplinary team approach was essential for implementing plant-based diets into clinical practice and achieving improved patient outcomes [32,33], particularly in conveying safe and consistent dietary messages. A concerted effort was also required to help harmonise differing nutritional philosophies and contradictory nutritional advice. This is especially important given the rapidly changing field, with recent developments suggesting that higher dietary potassium intake is not associated with hyperkalaemia or death in patients treated with haemodialysis [5]. When prescribing plant-based diet advice, additional attention to comorbid conditions is required. Confusion and miscommunication are commonly heightened in the case of comorbid conditions such as diabetes, which are prevalent in this population group [33], and can lead to increased feelings of anxiety amongst patients [33] as well as uncertainty around the value of diet in CKD management.

There is extensive research to support the need for individualised interventions for patients receiving dietetic care [34]. Dietitians in this study described the nuanced approach that may be beneficial when educating people with CKD about a plant-based diet. Approximately 25% of renal patients have limited health literacy [35] and high rates of cognitive impairment, including those in the pre-dialysis stages [36]. Despite this, nutrition resources for CKD management are often not designed to accommodate these deficits [37]. To help accommodate these challenges, dietitians in this study recommended using pictorial resources with limited text to aid comprehension of technical information. As recommended in previous research, strategies encompassing graduated goal-setting and collaborative decision-making [34] were also suggested to support patient adherence and inspire independence [38]. To overcome the sense of frustration described by patients with CKD when receiving didactic nutrition advice [2], the dietitians in the present study suggested an additional explanation of the benefits of plant-based diets, with explicit details on the anticipated benefits for the individual, were needed at the time of education. This may also help alleviate feelings of uncertainty that have been outlined in previous studies when patients are unclear of the reason for making dietary changes [39]. This idea to promote anticipated benefits is similar to findings from a review [40] in the field of diabetes that summarised the literature relating to the barriers and facilitators identified for implementing plant-based diets

There are several strengths and limitations to this research. The mixed-methods approach incorporates a survey and semi-structured interviews with two exclusive participant groups, enabling a rich collection of data. The use of open-ended and semi-structured questions in both the survey and interview allowed participants to articulate their perceptions to a greater extent and mitigates the risk of researcher bias. The major limitation of this research is the relatively small sample size for the survey. Furthermore, the perspectives gained may not represent all Australian renal dietitians or those working outside Australia. Hence, the findings offer general theoretical concepts that require further research to verify their applicability to other dietitians working in renal care.

## 5. Relevance to Practice

Interest in plant-based diets as a therapeutic option for CKD continues to grow globally, and recent studies have explored dietitians [41,42] and nephrology professionals’ [43] perceptions of plant-based eating. However, to our knowledge, this is the first study to provide explicit information on what might be required in practice to implement plant-based diets and strategies dietitians could use to support their patients with CKD to consume more plant foods.

Dietitians agreed that plant-based diets are beneficial for patients with chronic kidney disease. The successful implementation of plant-based diets was perceived to require extensive contact and education of patients in conjunction with ongoing support from a dietitian. In addition to educating patients, dietitians also need to consider buy-in from the MDT. Identified strengths of the hypothetical dietary prescription that are translatable to practice included shifting the focus of dietary advice to whole foods and overall healthy eating patterns rather than nutrients; positive framing of nutrition messages that encourage the inclusion of healthy foods; and practical supplementary resources such as recipes. Increasing knowledge about the benefits of the plant-based approach and starting with small incremental dietary changes were also recommended to increase patient acceptance.

## Figures and Tables

**Table 1:** Demographic characteristics of the study populations.

Characteristics	Interview (*n* = 11)	Survey (*n* = 35)
Gender (female, %)	11 (100%)	-
Age (range)	25–64	-
Years actively working as a renal dietitian (median-IQR)	9.17 (3.67–27.50)	7 (3–12.25)
Current employment status (Full time equivalent: median, IQR)	0.6 (0.3–1.0)	-
Practice setting		
Community settings	1 (9%)	-
Private practice	1 (9%)	-
Public health/hospitals	10 (91%)	-
Area of practice		
Early CKD	2 (18.2%)	11 (31.4%)
Pre-dialysis	7 (63.6%)	23 (65.7%)
Haemodialysis	8 (72.7%)	31 (88.6%)
Peritoneal dialysis	5 (45.5%)	20 (57.1%)
Renal transplant	4 (36.4%)	12 (34.3%)
Renal supportive care/palliative care	5 (45.5%)	13 (37.1%)
Other	0 (0%)	3 (8.6%)

‘-’ data not available for survey respondents. IQR, Interquartile range.

## Data Availability

The data presented in this study are not publicly available in accordance with the type of consent obtained about the use of confidential data.

## Supplementary Materials

The following supporting information can be downloaded at: https://www.mdpi.com/article/10.3390/nu14010216/s1; Item S1. Consolidated criteria for reporting qualitative studies (COREQ): a 32-item checklist; Item S2. Online survey questions; Item S3. Interview Guide; Item S4. Example of the seven-day food-target template; Item S5. Example of the foods swap list.
