# Supplementary material for: Acceptability of Plant-Based Diets for People with Chronic Kidney Disease: Perspectives of Renal Dietitians
Source: Nutrients. 2022 Jan 4;14(1):216. doi: 10.3390/nu14010216 (PMC8747619; doi:10.3390/nu14010216)
Supplement: Supplementary file 1 [file nutrients-14-00216-s001.zip › nutrients-1536671-supplementary.pdf]

Supplementary materials

Supplementary material – Item S1

Consolidated criteria for reporting qualitative studies (COREQ): a 32-item checklist
Developed from: Tong A, Sainsbury P, Craig J. Consolidated criteria for reporting qualitative research (COREQ): a 32-item checklist for interviews and focus groups. International Journal for Quality in Health Care. 2007. Volume 19, Number 6: pp. 349 – 357

No. Item	Guide questions/description	Reported
**Domain 1: Research team and reflexivity**		
Personal Characteristics		
1. Interviewer/ facilitator	Which author/s conducted the interview or focus group?	Mikaela Zuck & Jordan Stanford
2. Credentials	What were the researcher's credentials? E.g. PhD, MD	MK-Honours Research Student; JS-Australian Accredited Practising Dietitian and PhD student
3. Occupation	What was their occupation at the time of the study?	Honours and PhD Students
4. Gender	Was the researcher male or female?	Females
5. Experience and training	What experience or training did the researcher have?	Systematic training on qualitative methodologies and prior experience as research assistant/ research students on qualitative studies.
Relationship with participants		
6. Relationship established	Was a relationship established prior to study commencement?	Yes. Some participants may have been known professionally to the research group either through their affiliation from prior research studies or previous employment.
7. Participant knowledge of the interviewer	What did the participants know about the researcher? e.g., personal goals, reasons for doing the research	Participants were informed that researchers were undertaking this research as a part of their research/ course work.
8. Interviewer characteristics	What characteristics were reported about the interviewer/facilitator? e.g. Bias, assumptions, reasons and interests in the research topic	Participants were informed of the researchers' affiliations and interests. The research team were looking to commence a clinical research trial investigating the impacts of a plant-based dietary prescription with individuals diagnosed with stage 3-4 CKD in the near future.
**Domain 2: study design**		
Theoretical framework		
9. Methodological orientation and Theory	What methodological orientation was stated to underpin the study? e.g. grounded theory, discourse analysis, ethnography, phenomenology, content analysis	Qualitative thematic analysis by Braun & Clarke (2006) – applied inductively.
Participant selection		
10. Sampling	How were participants selected? e.g. purposive, convenience, consecutive, snowball	Convenience sample
11. Method of approach	How were participants approached? e.g. face-to-face, telephone, mail, email	Face-to-face (at the conference) or via email.
12. Sample size	How many participants were in the study?	Forty-six renal dietitians, 35 completed an online survey, while the remaining 11 participated in an in-depth interview.
13. Non-participation	How many people refused to participate or dropped out? Reasons?	Recruitment emails were sent via a professional email distribution network for renal dietitians in Australia. Five renal dietitians were invited at the Nephrology

		conference, of which all expressed interest. However, one individual who originally expressed interest could not participate due to scheduling conflicts.<br>Reasons for others who declined were unable to be acquired due to the nature of the recruitment method.
Setting		
14. Setting of data collection	Where was the data collected? e.g. home, clinic, workplace	Interviews were completed face-to-face or virtually, either via telephone or skype.
15. Presence of non-participants	Was anyone else present besides the participants and researchers?	No.
16. Description of sample	What are the important characteristics of the sample? e.g. demographic data, date	Australian dietitians (APD) provide dietary advice to individuals with CKD.
Data collection		
17. Interview guide	Were questions, prompts, guides provided by the authors? Was it pilot tested?	Interview questions were provided to participants during recruitment. Pilot testing and reflections on questions occurred during the first interview.
18. Repeat interviews	Were repeat interviews carried out? If yes, how many?	No.
19. Audio/visual recording	Did the research use audio or visual recording to collect the data?	Audio recording.
20. Field notes	Were field notes made during and/or after the interview or focus group?	Yes, some field notes were made during the interviews.
21. Duration	What was the duration of the interviews or focus group?	30-45 minutes.
22. Data saturation	Was data saturation discussed?	Yes.
23. Transcripts returned	Were transcripts returned to participants for comment and/or correction?	No.
**Domain 3: analysis and findings**		
Data analysis		
24. Number of data coders	How many data coders coded the data?	Two main coders. A third member of the research team coded a subset of transcripts.
25. Description of the coding tree	Did authors provide a description of the coding tree?	Yes.
26. Derivation of themes	Were themes identified in advance or derived from the data?	Derived from data using an inductive approach.
27. Software	What software, if applicable, was used to manage the data?	Dedoose 8.2.14
28. Participant checking	Did participants provide feedback on the findings?	No.
Reporting		
29. Quotations presented	Were participant quotations presented to illustrate the themes/findings? Was each quotation identified (e.g. ID)?	Yes.
30. Data and findings consistent	Was there consistency between the data presented and the findings?	Yes.
31. Clarity of major themes	Were major themes clearly presented in the findings?	Yes.
32. Clarity of minor themes	Is there a description of diverse cases or discussion of minor themes?	Yes.

Supplementary material – Item S2

Online survey questions

1. How long have you worked as a renal dietitian?

   ____

   ____

2. What is the main population(s) of people with chronic kidney disease (CKD) that you work with/have you worked with?
   a. Early CKD
   b. Pre-dialysis
   c. Haemodialysis
   d. Peritoneal Dialysis
   e. Renal transplant
   f. Renal supportive care/ palliative care
   g. Other: ____

   ____

3. The proposed renal diet plan aims to test the feasibility and impact of consuming 30 different plant-based foods over a week (7 days). Do you think this amount of plant foods for participants to consume is realistic? Please specify why in the comment box below.
   a. Yes
   b. No
   c. Unsure
   d. Why:

   ____

   ____

4. What barriers and/or challenges do you anticipate patients may encounter when trying to implement plant-based diets and achieve the target of consuming 30 different plant foods over a week (7 days)? You can answer more than one response.
   a. Perception of expense
   b. Cooking skills or preparation
   c. Previous education
   d. Food preferences
   e. Other:

   ____

   ____

5. Do you have any suggestions that could facilitate implementation and ensure adherence by people with CKD adhering to a plant-based diet or consuming 30 different plant foods within a week (7 days)? You can answer more than one response.
   a. Providing recipes
   b. Providing education
   c. Providing food items
   d. Other:

   ____

   ____

6. What aspects of a diet rich in plant-based foods for individuals with kidney disease would you be most cautious about as a renal dietitian? Please specify why in the comment box below.
   a. Prescribing more nuts and seeds
   b. Prescribing more whole-grain products
   c. Prescribing more dried fruit
   d. Prescribing more whole fruits
   e. Prescribing more vegetables, including legumes

   Other:
   ____
   ____

7. What support and/or resources would you like to have around diets rich in plant-based foods for people with CKD in your practice?
   ____
   ____
   ____
   ____

8. Do you have any additional comments/ suggestions
   ____
   ____
   ____
   ____

Supplementary material – Item S3

Interview Guide

Introductory questions (15 minutes)

Perceptions and experiences

- What barriers and/or challenges do you anticipate or have experienced when implementing plant-based diets with your patients?

- What are some of the enablers/solutions do you anticipate or have experienced when implementing plant-based diets with your patients?

Hypothetical prescription and accompanying materials (20-30 minutes)

Content

- How well do you think patients will understand the hypothetical dietary prescription and accompanying print resources? How do you think patients will feel about this diet (compared to current dietary prescriptions)?

- Which parts of the hypothetical dietary prescription and accompanying print resources do you like the best?

- Were there any aspects of the hypothetical dietary prescription and accompanying print resources that you do not like?

- How does the hypothetical dietary prescription and accompanying print resources differ from those you have used with patients in the past?

Practicability

- Do you think the amount of plant-based foods patients are required to eat was realistic?

- How do you think you would create a personalised meal plan with your patients using this approach?

- How likely do you think patients are to follow this diet?

- Do you anticipate any challenges in administering this hypothetical dietary prescription to your patients at your workplace? If so, how would you overcome them?

Future recommendations

- What aspects of the print resources would you change, and how would you improve them?

- What aspects of the hypothetical dietary prescription would you change? Do you have any suggested improvements to make it easier for patients to follow?

Supplementary material – Item S4

Example of the seven-day food target template

The term “serves" indicated under each food group (left-hand column) is used to represent the number of “food servings per day” to be consumed. Various versions of the template were created including an empty template, one-day pre-filled and three-day pre-filled template. Meal plans were modelled for different nutritional requirements based on energy intakes ranging from 5,500kJ to 12,000kJ. The meal plan below was modelled using the estimated nutritional requirements based on an average 85kg male adhering to the dietary guidelines for stage 3 chronic kidney disease [1]. Further adjustments may be required as the dietary guidelines continue to be updated in the future. The five bolded boxes under each day were provided as a visual aid to encourage/ prompt patients to select a unique plant food they hadn’t chosen previously within the last seven days. The accompanying 'food swap' list provided examples of what constitutes a food serving for each food group (Item S5, Supplementary Materials).

MY FOOD PLAN

**Food Groups**	Monday	Tuesday	Wednesday	Thursday	Friday	Saturday	Sunday
**Legumes/beans/ nuts/seeds** **1-2 serves**	15g mixed nuts						
**Vegetables** **6 serves**	½ cup cooked carrots<br>½ cup cooked broccoli<br>2 cup salad (leafy greens)<br>½ cup cooked green beans<br>½ cup sweet potato						
**Fruits** **2 serves**	1 pear<br>1 apple						
**Grains** **6 serves**	4 Weet-bix<br>1 cup brown rice<br>Unsalted popcorn<br>Plain wholemeal scone						
**Meat/Poultry/Fish** **180g**	40g shaved meat on sandwich (turkey/ chicken)<br>140g lean meat at dinner (beef)						
**Diary** **1 serve**	1 cup milk						
**Extras**	- 1 tbsp mayonnaise<br>- 2 tsp jam<br>- 2 tsp margarine or unsalted butter<br>- 2.5 tbsp. extra virgin olive oil<br>- 1 tsp honey						

**Please note that further changes have been made to the accompanying print resources following the feedback provided by the dietitians who participated in this study.*

Supplementary material – Item S5

Example of the 'foods swap list

Weights were expressed in cups or metric units such as grams (g). The listed plant foods were categorised into two separate columns according to potassium content, as summarised from a compilation of online renal resources and FoodWorks nutrient analysis software (Xyris Software, FoodWorks, version 10). Plant foods listed under the "Enjoy daily" column equated to ≤3mmol of potassium/serving. Plant foods listed under the "Enjoy a few times a week" column were > 3mmol of potassium/ serving. The rationale behind this was to allow additional flexibility for dietitians when educating and counselling patients, in case greater attention to controlling dietary potassium intakes may be clinically indicated. The intent was still to advocate for the inclusion of plant foods if desired by the patient (even foods with higher potassium content) but shift the focus of education on appropriate serving sizes and frequency of consumption, to prevent or manage hyperkalemia.

These drafted resources were designed to be used as a tool during consultations with patients. Therefore, interview participants were made aware that adaptations would be required in order to individualise it to each patient. For example, crossing out column headings and additional notes designed to help control dietary potassium intakes as it might not be relevant, or adapting suggested daily food servings to accommodate dietary preferences, allergies and intolerances, (i.e. vegetarian, nut allergies, lactose or gluten intolerances).

Servings for lean meat, poultry, fish and eggs were defined as the maximum total amount (in grams (g)) that can be consumed over a day. To make it easier for patients when trying to estimate portion sizes, it was explained to interview participants that servings would include the pre-cooked weight (i.e. raw lean beef mince) or the final weight if it is a 'ready to eat' product (i.e. shaved deli ham).

All bread, cereals, grains, and other products like pasta were counted in terms of grain foods. However, whole grains, wholemeal or high fibre, low GI varieties are preferred and encouraged over refined grains products.

MY SWAP LIST

All of the foods on this list = 1 SERVE

I can choose:
**____ Fruit ____ Vegetable ____ Grain ____ Legumes/ Nuts/ Seeds**
**AND *<u>180g</u>* Meat/Poultry/Fish/Eggs AND *<u>1</u>* serve of dairy (i.e. 1 cup/250g milk or 200g yoghurt or 2 slices/20g cheese)**

Plant-based food groups	Enjoy Daily	Enjoy a few times a week
**LEGUMES/BEANS/NUTS & SEEDS**		
	80g Tofu<br>1/2 cup Lentils (drained)<br>1/2 cup Chickpeas (drained)<br>75g Quorn<br>2 Tbsp Hummus	1/3 cup Baked beans<br>1/3 cup Bean mix
	15g Macadamia nuts (unsalted)<br>15g Pecan nuts (unsalted)<br>15g Hazelnuts (unsalted)<br>15g Almonds (unsalted)<br>15g Walnuts (unsalted)<br>15g Cashews (unsalted)<br>15g Peanuts (unsalted)<br>1.5 Tbsp chia seeds, sunflower seeds	
**VEGETABLES**		
**FREE VEGETABLES**<br><br>**1 serve = 1 cup raw**<br>**OR 1 serve = ½ cup cooked**<br><br>**Peel, dice and boil vegetables and discard the cooking water before eating*<br>**Drain all canned vegetables before eating*	Mushroom<br>Onion<br>Capsicum<br>Radish<br>Choko<br>Lettuce<br>Celery<br>Carrot<br>Bok choy<br>Eggplant<br>Asparagus<br>Bean sprouts	Spinach<br>Tomato<br>Turnip<br>1 Tbsp Tomato paste<br>1 cup vegetable juice (100%) or vegetable soups<br>Parsnip<br>¼ Avocado<br>Artichoke<br>Cauliflower<br>Brussel sprouts<br>Red cabbage

	Cabbage<br>Green beans<br>Zucchini<br>Watercress<br>Herbs and spices (i.e. garlic, parsley, dill, coriander)<br>Broccoli<br>Leek<br>4 slices Tinned beetroot (drained)<br>Silverbeet<br>Cucumber<br>Fennel	
**STARCHY VEGETABLES**	½ cup Canned corn<br>½ cup Baby corn<br>½ cup Peas	½ cup Sweet potato<br>½ cup Corn<br>½ cup Pumpkin<br>½ medium Potato
**FRUITS**		
**Drain all canned fruit before eating.*	1 medium Apple<br>1 Pear<br>¾ cup Grapes<br>250g Strawberries<br>1 cup Cranberries<br>¾ cup Blackberries<br>¾ cup Blueberries<br>¾ cup Boysenberries<br>¾ cup Raspberries<br>2 medium Mandarins<br>10 Lychees<br>1 ½ cups Watermelon<br>½ cup Rockmelon<br>1 ½ Grapefruit<br>1 Orange<br>1/3 Paw-Paw<br>1-2 Passion fruits<br>1 ½ cup Cherries<br>2 small Plums<br>½ cup Drained tinned peaches	1 small Banana<br>2 Tbsp Raisins<br>3 Dates<br>2 Nectarines<br>2 Star fruits<br>1 Nashi Pear<br>2 Kiwi fruit<br>1 Peach<br>1 small Mango<br>1 cup Papaya<br>½ cup Pomegranate<br>3 Dried figs<br>75g Custard-apple<br>2 Apricots<br>¾ cup mulberries<br>3-4 Prunes
**GRAINS**		
**BREADS**	1 slice of bread: multigrain, wholemeal, rye, raisin bread, sourdough, high fibre/low GI white varieties<br>1 small or ½ regular Wholemeal pita / ⅓ naan<br>1 small or ½ regular Wholemeal pita / ⅓ naan	1 plain scone

	½ Lebanese flatbread<br>1 Crumpet<br>½ English muffin	
**CEREALS**	2 Weet-Bix biscuits<br>½ cup Nutri-Grain, Puffed wheat, Guardian, Special K, Corn Flakes, Wheat Bran, Wheatgerm<br>¼ cup raw rolled oats<br>⅔ cup cooked rolled oats	⅓ cup Bran cereals: All-Bran/ Just Right/ Sultana Bran/ Muesli
**PASTA AND GRAINS**	½ cup cooked Pasta (all varieties)<br>½ cup cooked Noodles: rice, egg<br>½ cup cooked Rice: White/ Brown/ Basmati/ mahatma rice/ Doongara<br>⅓ cup cooked Polenta	⅓ cup cooked Barley<br>¼ cup Amaranth<br>½ cup cooked Quinoa
**CRACKERS AND BISCUITS**	4 Cruskits, Vita Weets<br>6 Salada, water crackers<br>2 Rice cakes<br>3 Saos<br>2 Plain biscuits: milk arrowroot	

References:

1. Ash S.; Campbell KL.; Bogard J.; Millichamp A. Nutrition prescription to achieve positive outcomes in chronic kidney disease: a systematic review. *Nutrients* **2014**;6, 416-451, doi:10.3390/nu6010416
